# Optimization of PCR conditions to amplify microsatellite loci in the bunchgrass lizard (*Sceloporus slevini*) genomic DNA

**DOI:** 10.1186/1756-0500-4-26

**Published:** 2011-01-31

**Authors:** Satya S Narina, Christian A d'Orgeix, Brian L Sayre

**Affiliations:** 1 Department of Biology, Virginia State University, Petersburg, VA-23806,USA

## Abstract

**Background:**

Microsatellites, also called Simple Sequence Repeats (SSRs), repetitions of nucleotide motifs of 1-5 bases, are currently the markers of choice due to their abundant distribution in the genomes, and suitability for high-throughput analysis. A total of five different primer pairs were optimized for polymerase chain reaction (PCR) to amplify microsatellite loci in total genomic DNA of bunchgrass lizards (*Sceloporus slevini*) collected from three sites in southeastern Arizona; the Sonoita Plain, Chiricahua Mountains and Huachuca Mountains.

**Findings:**

The primers used for current investigation were originally designed for the Eastern Fence Lizard (*Sceloporus undulatus*). Five primer pairs were selected based on annealing temperatures for optimizing the PCR conditions to amplify with bunchgrass lizards. Different concentrations of DNA and annealing temperature were optimized. While keeping other reagents constant, a DNA concentration, 37.5 ng in the final reaction volume and PCR conditions of an initial denaturation of 94°C for five minutes, an annealing temperature of 55°C and final extension of 72°C for four minutes gave the best amplification for all the primer pairs.

**Conclusions:**

Modifying the standard protocol for annealing temperatures and final extension time increases the success of cross amplification of specific microsatellite loci in the bunchgrass lizard. A loading volume of 5 ul DNA at a concentration of 10 ng/ul and a 2% agarose for gel electrophoresis were observed the best for cross amplification of selected five primer pairs on bunch grass lizard.

**Trial Registration:**

The research was conducted with Arizona Game and Fish Department scientific collecting permits SP565256, SP657407 & SP749119 to Dr. Christian A d'Orgeix.

## Background

Small, isolated or bottlenecked populations suffer reduced genetic diversity increasing their potential for extinction [1]. Terrestrial "islands" such as mountain tops can function to isolate populations and reduce or halt gene flow between populations [2,3]. Slevin's bunchgrass lizard, *Sceloporus slevini *[4] inhabits both disjunct mountain grasslands in southeastern Arizona, southwestern New Mexico, the Sierra Madre Occidental of Mexico [5-7] and lower elevation grasslands in the Sonoita plain and Canelo grasslands in Arizona [7,8]. The disjunct population distribution of *S. slevini *and the recent bottleneck of a low elevation population [8] make it an ideal species to test a model of reduced genetic diversity within disjunct or bottlenecked populations.

A necessary preliminary step in examining the genetic characteristics of this species is to develop molecular species specific markers. Species-specific microsatellite markers [9] can be used to elucidate gene flow patterns and genetic diversity studies [10]. The SSR based microsatellite markers have been developed for a large number of plant [11,12] and animal [13] species and are increasingly being used for ascertaining germplasm improvement. The primer sequences used for the current project were originally developed for the Eastern Fence Lizard [14].

An important limitation, however, regarding use of microsatellites for polymorphism or genetic diversity studies is the prior need for optimization of PCR conditions for each SSR marker [15]. The Genomic DNA sequence variation between two different species might cause the variation in cross amplification success as the primers might identify a different region in the species of interest from the expected region. Required amplification may not be obtained using the recommended protocol due to involvement of many factors such as different types/brands of thermocyclers, reaction components, or even minor differences in thickness of walls of PCR tubes [16]. In addition, the quality and quantity of template DNA obtained with different DNA extraction protocols may also affect the PCR results.

The primer sequences selected for the current study were originally developed for *S. undulatus*, [14]. These primers were used to amplify the genomic DNA of *S. slevini *following the protocol published [14]. The cross amplification was not satisfactory even though *S. slevini *is a congener of *S. undulatus*. In order to reduce the time and cost involved in generation of ESTs and development of new primers from *S.selvini*, further protocol standardization was undertaken [16] by changing the annealing temperatures. PCR conditions were optimized for 5 SSR markers in *S. slevini*. In addition to PCR, agarose gel concentrations and sample loading volume were also optimized for clear visibility of microsatellites.

## Results

**Figure 1:**
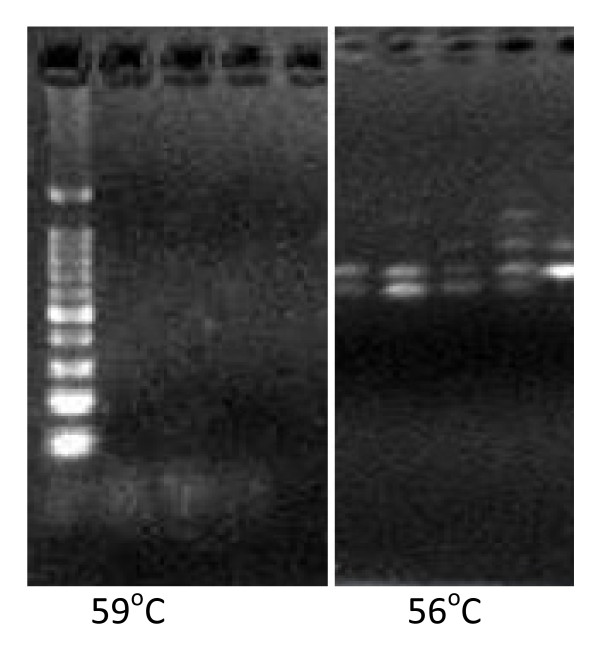
**Illustration of failure to amplify at 59 and 56°C**.

**Figure 2:**
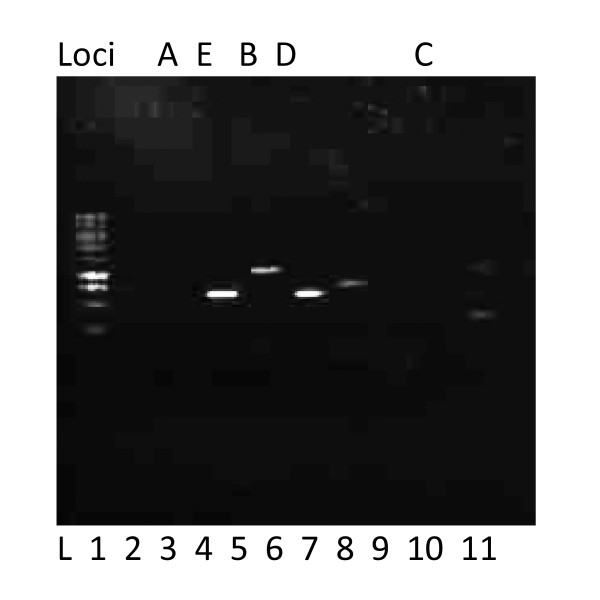
**Amplification (at 55°C annealing temperature) products of genomic DNA of Slevin's bunchgrass lizard (*S. slevini*) sample H13 on 2% agarose gel ran for one hour at 100 volts using 10 markers (1-10) and ladder (L); Lane1: Ladder; Lane2 -4,9 and 10: Empty; Lane 5: A; Lane 6: E; Lane 7: B; Lane 8: D; Lane 11: C**.

**Figure 3:**
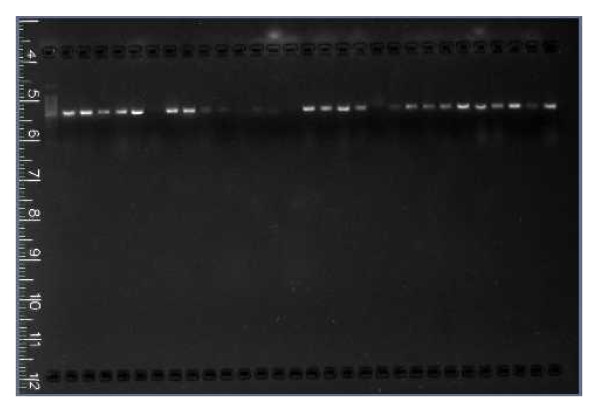
**Amplification (at 55°C annealing temperature) products (500 bp) of genomic DNA (5 ul) of bunchgrass lizard sample numbers 17 to 46 on 2% agarose gel ran for 30 minutes at 100 volts using marker Sun_011**.

The PCR conditions standardized for microsatellite amplification in this study were an initial denaturation at 94°C for five minutes; 35 cycles of denaturation (94°C) for 30 seconds, annealing (55°C) for 30 seconds, extension (72°C) for one minute followed by final extension at 72°C for four minutes. The current study achieved excellent cross amplification performance using the standardized PCR conditions and was visualized on simple agarose gel electrophoresis, though the preferred method was the standard DNA sequencing gel electrophoresis. Previous studies have indicated that the optimal annealing temperature was 58-60°C (Ta ≥ 56°C, [14]). However the PCR of selected markers at these conditions failed to amplify 45 out of the 50 samples tried. A series of PCRs were performed using these primer sets at multiple temperatures Viz., 53°C, 54°C, 55°C, 56°C, 57°C, 58°C, 59°C, 60°C and 61°C (Figure 1). The PCR products were not able to visualize on 1 to 1.5% agarose gels as solid bands. A series of fragments or a shadow of the band was observed instead of the solid single band. It was determined that it was necessary to electrophorese the samples on a 2% agarose gel inorder to eliminate the smaller fragments. The best amplification conditions for successful amplification of all the loci were at the lower annealing temperature of 55°C (Figure 2) and visualized on 2% agarose gel. The figures taken at annealing temperatures of 56°C and 59°C were also included in Figure1 to visualize and compare the improvement at 55°C in Figures 2 and 3.

The sequencing gel wasn't used to visualize the microsatellite fragments as the objective of the current project is to test the cross amplification of primers. As the primers are successfully cross amplified, the ongoing project will use these primers to screen the rest of the population and observe the diversity.

There were 50 individuals scored for 5 markers (Table 1). Not all the individuals amplified for all the five markers. There are null alleles marked as 0 for each marker against each individual in table 1. Individual H10 has no alleles for all the five loci tested. Only seven individuals are amplified at loci (marker) Sun_009 out of 50 tested. Among all the five loci tested, Sun_007, Sun_011 and Sun_012 are amplified in majority of the individuals. The loci Sun_011 and 012 are deviating from the expected size of 256 and 248 respectively to 500 bp and 600 bp in our study 9 (Table 2).

**Table 1:** Cross Amplification using five markers for 50 collections (1-Present,0-Absent,- unknown) at the Department of Biology, V.S.U., Petersburg.

		Markers				
		
Sample. No.	Name of the collection	Sun_007	Sun_009	Sun_010	Sun_011	Sun_012
1	A1	-	1	0	1	1

2	A2	0	0	0	1	1

3	A3	0	0	0	1	1

4	A4	1	1	-	1	1

5	A5	1	0	0	1	1

6	A6	0	0	-	0	1

7	H1	0	-	0	0	1

8	H2	0	0	0	1	0

9	H3	0	-	0	1	1

10	H4	1	1	0	0	1

11	H5	0	0	0	1	0

12	H6	1	0	0	1	-

13	H7	1	1	0	1	1

14	H8	1	0	-	1	0

15	H9	0	0	0	1	0

16	H10	0	0	0	0	0

17	H11	-	0	0	1	0

18	H13	-	1	1	1	1

19	H14	-	1	0	1	-

20	H15	1	0	0	1	1

21	H16	1	-	0	1	0

22	H17	0	0	0	0	0

23	C1	1	0	0	1	1

24	C2	1	-	0	1	1

25	C3	1	0	-	-	-

26	C4	1	0	1	-	0

27	C5	1	0	1	-	0

28	C6	1	0	1	-	0

29	C7	1	0	1	-	0

30	C8	1	0	1	0	0

31	C9	1	-	1	1	0

32	C10	1	0	1	1	1

33	C11	1	0	1	1	-

34	C12	1	0	-	1	-

35	C13	1	1	0	0	1

36	C14	1	0	-	-	-

37	C15	1	-	-	1	1

38	E1	1	0	0	1	-

39	#1	1	0	0	1	1

40	#2	1	0	0	1	1

41	#3	1	0	0	1	1

42	#4	1	0	-	1	1

43	#5	1	-	0	1	1

44	#6	1	0	0	-	-

45	#7	1	0	0	1	1

46	#8	1	0	0	1	-

47	#9	1	0	0	1	-

48	#10	1	0	-	1	-

49	#11	1	0	1	0	-

50	#13	1	-	1	1	-

(A-Appleton; H-Huachuca; C-Chiricahua; E-East France)

**Table 2:** 5 SSR markers with their observed and expected size of PCR products

Code to the marker	Locus Id	Observed size (bp) of the PCR product (Range) on 2% agarose gel	Size expected
A	Sun_007	300-400	317

B	Sun_009	300-400	453

C	Sun_010	200	259

D	Sun_011	500	256

E	Sun_012	600	248

## Discussion

Though the SSR primer sets were selected from *S. undulatus*, a relative of *S. slevini*, it was necessary to optimize the PCR conditions for *S. slevini *in order to produce readable results. The current study reveals that it is critical to optimize the reaction conditions prior to large-scale application of each locus, such as phylogenetic/genetic diversity studies. Interestingly, as shown in Figures 2 and 3, a lower annealing temperature provided more specific amplification of the locus. The reason for this might be primer mismatches at higher temperatures [17] as the primers were originally created for a related species. At elevated temperatures the nucleation of the primer hybridization becomes more difficult, thus, the nonspecific target sites, with some mismatched base pairs, compete with the specific target sites for the primer hybridization. The ratio of primers binding to the correct sites decreases allowing mismatched priming of the polymerization. Once the mismatched priming occurs, extension is also faster at higher temperatures, and thus, there will be promotion of a nonspecific polymerization reaction. However, at 1 or 2°C lower annealing temperatures, nucleation would take place easily and there will be no or little competition of nonspecific sites with a couple of mismatches for the correct target sites [18].

The current investigation also observed the primers tested were all monomorphic on all the individuals tested except for H13. This could be due to their same origin or proximity within the taxon even though they were collected from two different elevation points/geographical locations. The phylogenetic studies of lizard [19] revealed a general pattern of strong character support for the monopoly of species groups and weak support for the relationship between them occurs in the trees from the molecular, morphological and combined data sets which might be due to the rapid speciation.

We found that the optimal temperature for cross hybridization of the primer sets for *S. slevini *was lower than the optimal temperature for the original species (*S. undulatus*). This work confirmed that microsatellite primers created for one species can be used in another species with optimization of the primer sets as done in several organisms previously [20,14]. By using this protocol, remaining markers will be studied and will be used to characterize different populations of bunchgrass lizard and to understand the evolutionary similarities between the populations.

## Conclusions

The current study standardized a PCR protocol to amplify genomic DNA of *S.slevini *using the markers developed for *S. undulatus*. By increasing or decreasing the annealing temperatures and final extension time, the success of cross amplification of specific microsatellite loci increased markedly. The five markers tested were monomorphic in the bunch grass lizard. This is preliminary effort in bunchgrass lizard to generate markers due to non availability of funds and high throughput equipment in the lab. Out of the five, the loic Sun_007, Sun_011 and Sun_012 were usable for future screening as the frequency of null alleles was very low among the population tested. Further, these PCR products need to be sequenced to know the exact size of the amplified loci. These loci will be used to examine models of evolutionary history, gene flow patterns and inter and intra-population differences in *S. slevini*.

## Methods

Research was conducted under Arizona Game and Fish Department scientific collecting permits SP565256, SP657407 & SP749119 to Christian d'Orgeix. All applicable institutional and international animal care guidelines were followed.

Tissues from *S. selvini *were collected from two high elevation populations (n = 16, Huachuca Mts and n = 15, Chiricahua Mts) and one low elevation population (n = 6, Appleton-Whittell Research Ranch, Elgin) in Arizona in 2006 and 2007. The lizards were caught by hand and approximately 2-4 mm of their tail tips were removed using tweezers. The lizards were then released at the site of capture. Besides these there were 13 collections in the department without geographical location information, also included for current investigation. Tissues were placed in 90% isopropyl alcohol for 1-2 months. DNA was extracted using DNeasy tissue kit [21]. Approximately 25 mg of tissue (0.5 cm length of tail) was used for DNA isolation using the protocol [21] and the extracted DNA samples were stored at -20°C for future use.

DNA was quantified at 260 nm wavelength by using Smart spec, 3000 spectrophotometer (http://www.bio-rad.com/LifeScience/pdf/Bulletin_2634.pdf) and dilutions of known concentrations were prepared. Absorbance ratios of the extracted genomic DNA at 260 nm and 280 nm were ranging from 1.34 to 2.00. Quality of DNA was also confirmed by electrophoresing DNA on 1.1% agarose gel. Out of total 39 SSR markers previously reported [14] in *Sceloporus undulatus*, only 10 markers were tested in this study.

The PCRs were performed in the absence of oil for 50 μL of reaction volume using simple PCR reaction in BioRad's "i"cycler. Reaction mixture included template DNA (10 ng) solution, forward and reverse primers (0.15-0.30 μM), and Platinum PCR Supermix (Invitrogen Corporation). The cycling reactions were conducted for 5 min at 94°C, followed by 35 cycles of 30 sec denaturation step at 94°C, 0.5-1.0 min primer annealing step at varying temperatures of 53°C, 54°C, 55°C, 56°C, 57°C, 58°C, 60°C and 61°C, and 4 min extension step at 72°C. The amplification of the markers were detected by running the amplicons on 2% agarose gel in 1X TBE buffer, stained with ethidium bromide (500 ng/mL), visualized and photographed using VersaDoc Imaging system Model 4000 (BIORAD suppliers). All gels were run at 106V constant power supply for 1-2 hours. We tried loading different volumes of DNA (1-5 ul) with different concentrations from 5 to10 ng/ul. The gel images were clear with solid bands at 10 ng/ul concentration and loading volume of 5 ul.

The Primer pairs Sun_019, 020, 030, 033 and 038 were not amplified on bunchgrass lizard samples using published protocol as well as our standardized protocol at 55°C annealing temperature (Figure 2). Therefore only five primer pairs were selected for current diversity studies and the observed and expected band sizes for each locus were given in Table 2.

## Competing interests

The authors declare that they have no competing interests.

## Authors' contributions

SN designed the experiment, carried out the molecular genetic studies and drafted the manuscript. CD and BS participated in preparation of the manuscript. All authors read and approved the final manuscript.
